# Carbon and Tin-Based Polyacrylonitrile Hybrid Architecture Solid Phase Microextraction Fiber for the Detection and Quantification of Antibiotic Compounds in Aqueous Environmental Systems

**DOI:** 10.3390/molecules24091670

**Published:** 2019-04-28

**Authors:** Sandip Mondal, Jialing Jiang, Yin Li, Gangfeng Ouyang

**Affiliations:** MOE Key Laboratory of Bioinorganic and Synthetic Chemistry/KLGHEI of Environment and Energy Chemistry, School of Chemistry, Sun Yat-sen University, Guangzhou 510275, China; sandip1706@yahoo.com (S.M.); 18826133611@163.com (J.J.); yinli5@mail2.sysu.edu.cn (Y.L.)

**Keywords:** antibiotics, composite materials, solid-phase microextraction, LC-MS/MS, water matrices

## Abstract

In this study, the detection and quantification of multiple classes of antibiotics in water matrices are proposed using a lab-made solid phase microextraction (SPME) fiber coupled with high-performance liquid chromatography-tandem mass spectrometry (LC-MS/MS). The lab-made fiber was prepared using a graphene oxide (G), carbon nanotubes (C), and tin dioxide (T) composite, namely GCT, with polyacrylonitrile (PAN) as supporting material. The detected antibiotics were enrofloxacin, sulfathiazole, erythromycin, and trimethoprim. The custom-made fiber was found to be superior compared with a commercial C18 fiber. The excellent reproducibility and lower intra-fiber relative standard deviations (RSDs 1.8% to 6.8%) and inter-fiber RSDs (4.5% to 8.8%) made it an ideal candidate for the detection of traces of antibiotics in real environmental samples. The proposed validated method provides a satisfactory limit of detection and good linear ranges with higher (>0.99) coefficient of determination in the aqueous system. Application of the method was made in different real water systems such as river, pond and tap water using the standard spiking method. Excellent sensitivity, reproducibility, lower amount of sample detection and higher recovery was found in a real water sample. Therefore, the extraction method was successfully applied to the detection and quantification of multiple classes of antibiotics in different aqueous systems with satisfactory results.

## 1. Introduction

Antibiotics are a kind of antimicrobial substances produced from natural (bacterial or fungal source), semisynthetic or synthetic sources that possess the intrinsic ability of inhibiting the growth of or killing microbes [1]. Since the 1930s, antibiotics were used to treat and prevent human and veterinary animal infections and later to promote growth in livestock, poultry breeding, and aquaculture [2]. Therefore, it is not a surprising fact that they can be detected in different water bodies around the world up to µg level per liter due to the direct discharge of expired or unused medicines, metabolized drugs, the low removal efficacy of wastewater treatment plants for this class of substances and agricultural runoff [3,4,5]. The half-life of antibiotics is from around hours to 100 days [6], although antibiotic residues can be considered as a persistent organic contaminant in environment owing to their regular and widespread use and continual emissions [7]. Throughout the globe, the presence of antibiotics has been reported on groundwaters, surface waters and sediments [8,9,10,11,12,13]. Even at very low concentration antibiotics can exert adverse effects on ecology and human health [12,13]. The main side effects of antibiotics are the appearance of antibiotic-resistant pathogens carrying antibiotic-resistance genes and inhibition of the growth of aquatic organisms [14,15]. The forming antibiotic-resistant genes could invade into the human body via direct contact or food chain through horizontal gene transfer mechanism, and further antibiotic resistance causes the failure of the treatment and even death [16,17]. Therefore, monitoring and assessment of the antibiotics in a real water system is the need of this hour.

**Figure 1:**
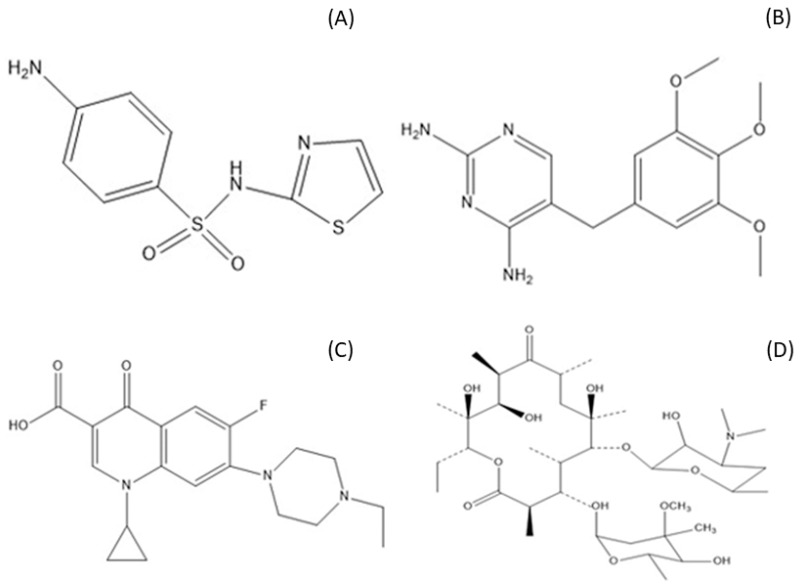
Chemical structures of (**A**) sulfathiazole (STZ), (**B**) Trimethoprim (TMP) (**C**) enrofloxacin (ENF) and (**D**) erythromycin (ERM).

Some of the important and commonly found antibiotic residues in the environment are erythromycin, trimethoprim, enrofloxacin, and sulfathiazole. Erythromycin belongs to the macrolide antibiotic class. It can be used to treat eye infections caused by bacteria [18]. Trimethoprim is a synthetic dihydrofolate reductase inhibitor used to treat certain kind of bacterial infections such as urinary tract infections [19]. Enrofloxacin is a synthetic, broad-spectrum antibiotic that belongs to the fluoroquinolones class. This antibiotic is used to treat bacterial infections in veterinary animals [20]. Sulfathiazole is a short-acting sulfonamide used primarily in veterinary medicine [21]. The structures of these antibiotics are presented in Figure 1.

Monitoring antibiotics in real water samples is a real challenge due to the ultra-low concentration of the target compounds, the diversity of compounds and the high complexity of the sample matrix [22]. Therefore, before using chromatographic or hyphenated techniques sample enrichment is an essential step. The most common sample enrichment techniques to detect antibiotic residues are liquid-liquid extraction (LLE) [23,24], dispersive liquid-liquid microextraction [25,26], solid-phase extraction [27,28], solid-phase microextraction [29,30,31], pressurized liquid extraction [32,33], matrix solid-phase dispersion extraction [34,35], and the quick, easy, cheap, effective, rugged, and safe (QuEChERS) method [36,37]. Among these methods, SPME has gained popularity owing to its superior features such as low cost, high extractability, almost solventless uses and easy to operate technique. Previously, SPME has been applied successfully to the preconcentration of several trace antibiotic substances in water samples.

To detect several classes of an antibiotic mixture in a water environment is of prime importance that can be seen by published papers utilizing different methods. The abovementioned classes of drugs were detected individually or in combination with other classes in water samples. However, the simultaneous detection of ERF, STZ, ETM, and TMP by using SPME fiber was not reported so far.

Selection of a proper adsorbent material is of prime importance in SPME fibers to detect antibiotic compounds in water systems. In this respect, graphene is a carbon-based single-atomic-layer two-dimensional material. With different oxygen-containing functional groups, such as hydroxyl, carbonyl, carboxyl and epoxy groups on its surface and the presence of a delocalized plane π-electron system it might form strong π-π bonds utilizing π–π electron donor-acceptor interactions which makes it an ideal candidate for the detection of antibiotics [38]. Tin dioxide is a chemically stable, non-toxic, low cost, and possess high active surface area and surface to volume ratio [39]. Carbon nanotubes are an allotrope of carbon with a cylindrical nanostructure. Carbon nanotubes have a high affinity for polycyclic compounds, π–π stacking capability, large surface area, and high surface that make them ideal to detect antibiotics [40]. Using multiwalled carbon nanotubes (MWCNTs) rather than single-walled carbon nanotubes (SWCNTs) offers an advantage concerning adsorption capacity due to their concentric layers of graphene [40]. Based on the favorable detection capability of GO, carbon nanotubes and SnO_2_, this work synthesized a composite material GCT along with biocompatible PAN that helps to stick the composite on the surface of quartz fibers to detect four classes of antibiotic in aqueous solutions. 

In this present work, an analytical method based on SPME was developed to detect multiple classes of antibiotics in water. A novel and sensitive composite SPME fiber was synthesized and thoroughly characterized. The optimized extraction parameters of antibiotics from the aqueous medium were assessed and our fiber was confirmed to be much more efficient than commercial graphene, carbon nanotubes, tin dioxide, C18, polydimethylsiloxane (PDMS), and acrylate fibers. Finally, the fiber was successfully applied to detect multiple classes of antibiotic in different real aqueous environmental system.

## 2. Results and Discussion

### 2.1. Characterization of GCT Hybrid Architectures

The real challenge to bind the GCT compound onto the quartz fiber surface was solved by choosing appropriate gluing materials. The gluing material should be biocompatible and should not cover the GCT binding surface area [41]. PAN (dissolved in dimethylformamide (DMF) 1:10, *v*/*v*), was chosen as an auxiliary material due to its acid resistance and biocompatible nature [42,43,44]. After the third dip coating, the fiber surface was cured at 30 min for 120 °C; thus, the DMF was evaporated, and a porous matrix was formed on the surface of the GCT fiber. Thus, PAN not only helps to glue the GCT material but also helps form porous structures and thus provides dual characteristics [29,41].

**Figure 2:**
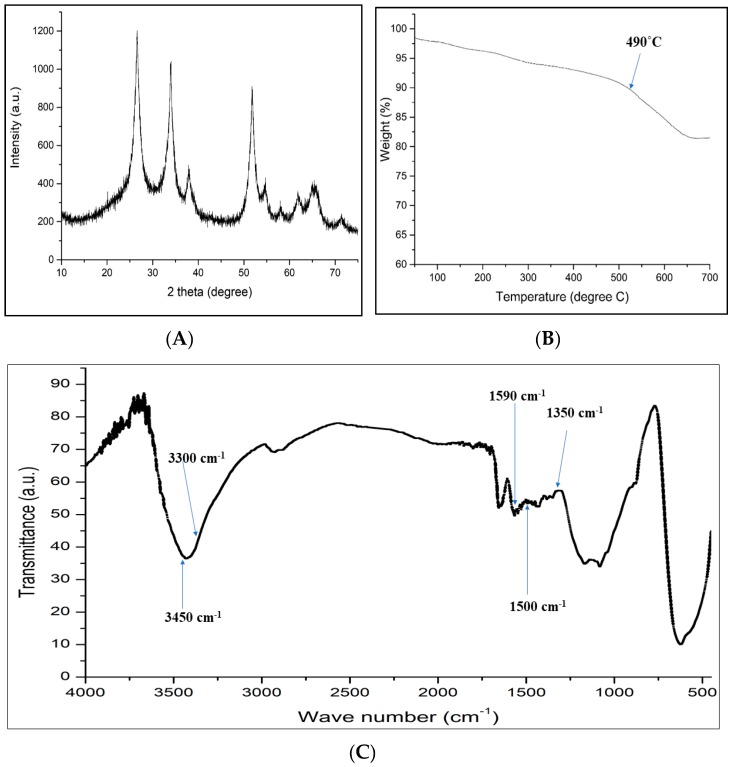
Image of (**A**) XRD, (**B**) TGA, and (**C**) FTIR images of GCT.

The XRD data was fitted using the JADE 6 software (6.0, Jade, Livermore, CA, USA) and the major diffraction peak was revealed to correspond to crystalline tetragonal rutile like SnO_2_ (JCPDS Card No. 41–1445, space group P42/mnm, a0 = b0 = 4.738 Å, c0 = 3.187 Å, alpha = beta = gamma = 90.0, volume 71.6, density 7.02). This observation (Figure 2A) was similar to the literature, thus confirming the formation of the desired compound [45].

Thermal gravimetric analysis (TGA) indicates the mass change of a material, either as a function of increasing temperature with constant heating rate, or as a function of time with constant temperature and consecutive mass loss, in an atmosphere of nitrogen, helium, air, other gas, or in a vacuum. TGA of the composite GCT material performed up to 700 °C under air indicated only one main decomposition events namely at 490 °C. From TGA analysis (Figure 2B), it is cleared that the material is stable up to 490 °C in air, confirming its higher thermal stability.

Potassium bromide (KBr) was combined with the powder to form a pellet which spectrum was recorded by Fourier Transform Infrared spectroscopy (FT-IR) to detect the functional group of the powder. Peaks at 3300 cm^−1^ and 3450 cm^−1^ represent asymmetrical and symmetrical vibrations of amine groups (Figure 2C). The peaks at 1590 cm^−1^ and 1350 cm^−1^ were ascribed to the N-H bending vibration and C-N stretching of aromatic amines in the GCT skeleton, respectively. The peaks at 1500 cm^−1^ correspond to the -(O-C-O)- stretching vibration.

The zeta potential was found to be −20.28 mV with mobility 1.51 (μ/s)/(V/cm) at pH 7.4 when dispersed in water using the Smoluchowski model (Figure S1A). The effective particle diameter of GCT was estimated to be 445.62 nm, polydispersity 0.216 and diffusion coefficient 1.103e^−8^ cm^2^s^−1^ (Figure S1B).

**Figure 3:**
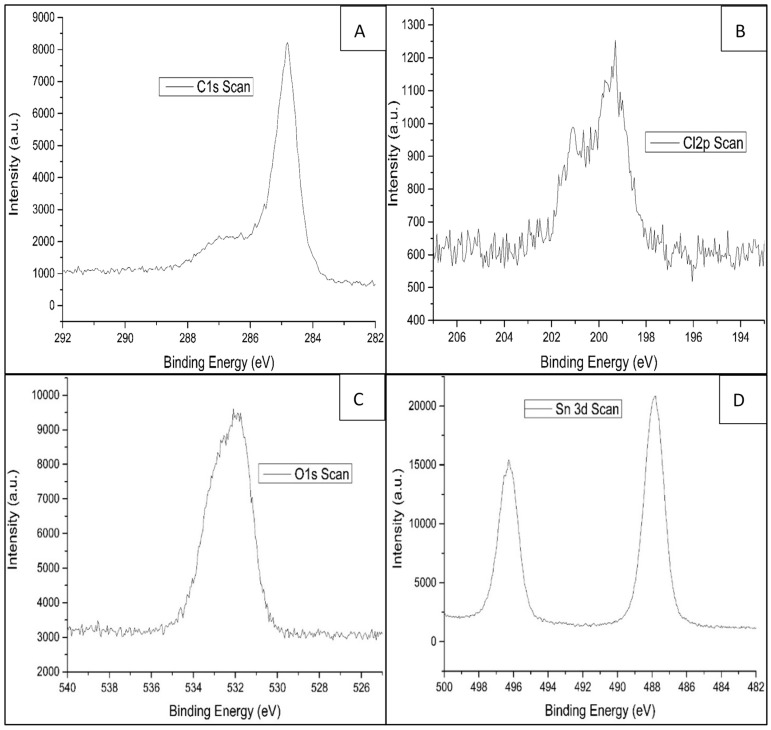
XPS spectra of (**A**) carbon, (**B**) chlorine, (**C**) oxygen, and (**D**) tin.

X-ray photoelectron spectroscopy (XPS) was used to confirm the elemental composition of the materials at the parts per thousand level. The spectra were obtained by irradiating the surfaces of powder with an X-ray beam, and the kinetic energy of the electrons released from the 0 to 10 nm-deep surfaces of the powder was analyzed. XPS data of the powder confirms the presence of carbon, chlorine, oxygen, and tin (Figure 3A–D).

The peaks located in the C, O, and Sn core level regions could be assigned to C 1s, O 1s, and Sn 3d, Sn 3p, and Sn 4d, respectively (Figure S2). The deconvoluted C 1s spectrum of GCT (Figure 3A) reveals four types of carbon bonds: C=C/C−C (284.7 eV), C−O (hydroxyl and epoxy, 286.6 eV), C=O (carbonyl, 287.5 eV), and O−C=O (carboxyl, 288.7 eV).

**Figure 4:**
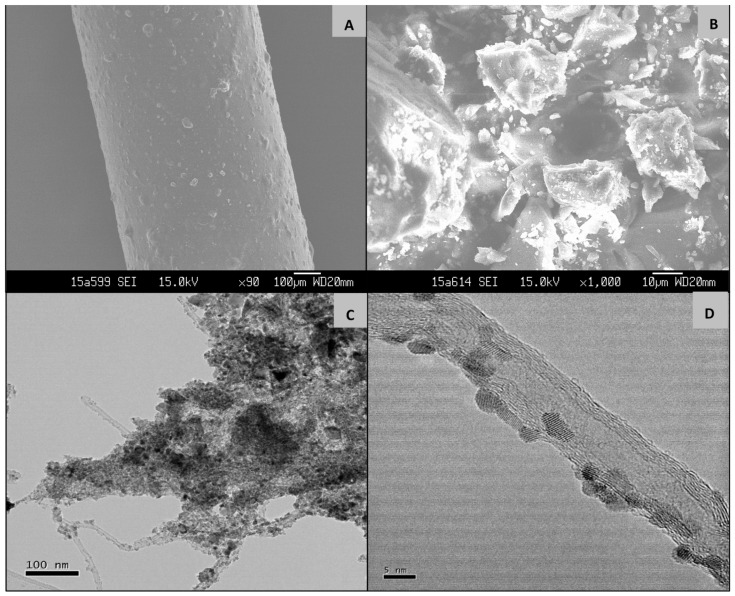
SEM images of GCT (**A**) fiber, (**B**) powder and TEM images of (**C**,**D**) GCT powder.

Scanning electron microscopy (SEM) analysis was carried out to examine the surface texture of GCT-coated SPME fiber. The uniform distribution of GCT material on the surface of the fiber helps to extract analytes efficiently (Figure 4A). Uniform thickness of the fiber is an important parameter to ensure the uniform extraction of analytes on the fiber surface and sensitivity of the fiber. After dipping the fiber into the PNA coating, the thickness was found to be 60 µm. The fiber diameter with and without material was 720 µm and 660 µm respectively. The SEM images of GCT powder were found to be porous, uneven and irregular shaped (Figure 4B).

The transmission electron microscopy (TEM) images of the GCT compound are shown in Figure 4C,D where the high-resolution TEM (HRTEM) confirms the presence of SnO_2_ NPs. The size of the NPs was 0.35 nm with 110 planes and 0.27 nm with 101 planes. The selected area electron diffraction (SAED) pattern additionally confirmed the presence of concentric circles and thick diffuse rings (Figure S3) were to be the graphene/CNTs and polycrystalline SnO_2_ species, respectively.

### 2.2. Cost Analysis of SPME Fiber Preparation

The cost involved in the preparation of SPME fiber through different steps, such as the cost of chemicals, equipment used, fiber cost, etc., is one of the vital facts to be taken into account to make the fiber cost-effective for its implementation in a large-scale application. Cost analyses of the preparation of SPME fibers employed for the detection of analytes are still scarce in the literature. The cost break-down for each step and the total cost for the preparation of SPME fiber in Chinese Renminbi (RMB) has been calculated in a stepwise fashion to give an idea of the approximate cost for the present investigation (Table S1). The approximate cost of an SPME fiber is found to be 6.53 RMB or 0.97 USD, which is much more inexpensive than a commercial fiber (1000 RMB or 149 USD).

### 2.3. Optimization of the GCT-PAN Hybrid Architecture Fiber Coating in Water for Antibiotics (ABs) Extraction

In order to obtain the highest extraction of ABs from water, single-factor tests were performed to optimize different parameters such as extraction time, agitation speed, salt concentration, desorption time and solvent system for extraction and desorption. A mixture of four antibiotic compounds was spiked with a concentration of 100 µg L^−1^ in the optimization experiment. For each of the parameters, three parallel experiments were carried out.

#### 2.3.1. Extraction Time

**Figure 5:**
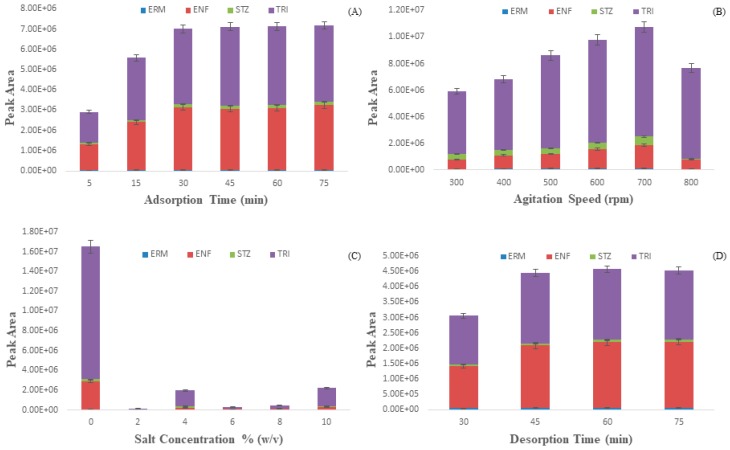
Effect of (**A**) extraction time (min), (**B**) agitation speed (rpm), (**C**) salt conc.% (*w*/*v*) and (**D**) desorption time to extract Antibiotics from water.

The extraction efficiency usually varies with extraction time as SPME is a time-dependent process where equilibrium is attained at the highest point between the donor and acceptor phases. Extraction time experiments were carried out from 5 to 75 min under constant experimental conditions to get the highest extraction efficiency. From Figure 5A, it was seen that the peak area of the analytes increased with increasing extraction time from 5 to 30 min. After 30 min, there is a negligible effect on the extraction efficacy. This phenomenon can be explained by the fact that at the beginning the GCTs active sites were vacant and the solute concentration was higher, thus attachment of AB molecules on the GCT surface was increased. After a certain period, antibiotic molecules attach on all the empty places of GCT thus gave an equilibrium point. At the equilibrium point, the attachment and detachment of ABs on the surface of GCT were comparable [46]. Thus, a contact time 30 min was chosen as an optimum extraction time for further study.

#### 2.3.2. Agitation Speed

One of the critical factors of SPME is agitation speed. Higher agitation speeds can accelerate the transfer of analyte from aqueous samples to the coating while reducing the thermodynamic equilibrium time. The effect of agitation speed was examined in the range of 300–800 rpm. It is observed from Figure 5B, that increasing the agitation speeds from 300 to 700 rpm resulted in an increase in extraction efficiency, and 700 rpm was considered the best agitation speed for the rest of the study. Further, an increase in agitation speed beyond 700 rpm resulting in a decline of extraction efficiency due to the vigorous collision of the solution thus the formation of bubbles ultimately affecting the transfer of the analyte [47,48], so finally, 700 rpm was chosen for further studies.

#### 2.3.3. Salt Concentration

The extraction efficiency of GCT was affected by salt concentration due to salts’ salting-in and salting-out effects. While salting-in decreases the extraction efficiency, salting-out enhances the extraction efficiency [49,50]. The salt concentration was varied in the range of 0.0–10.0% (*w*/*v*) with intervals of 2.0% (Figure 5C) while the other parameters (extraction time, 30 min; agitation speed, 700 rpm; desorption time, 60 min) were kept constant. The results evidenced that the addition of salt did not help ABs extraction due to the salting-in effect, so in the following experiments, no salt was added.

#### 2.3.4. Desorption Time and Solvent System

Complete desorption is a necessary step for method sensitivity and cleansing of the fiber; therefore, desorption time is an essential parameter for the extraction of ABs. This experiment was performed to make sure no carryover (Figure S4A) was detected after fiber desorption [51,52]. Desorption time in the range of 30–75 min was investigated. Figure 5D indicated that the peak areas increased as desorption time increased from 30 to 45 min, and further time leads to almost the same amount of peak area. Thus, 45 min was selected as the optimum desorption time for the subsequent studies.

The solubility of an antibiotic mixture in a suitable desorption solvent is an important extraction parameter as complete desorption means higher reproducibility and lesser carryover of the proposed methods [53]. In the laboratory, the two most superior solvent systems to dissolve organic compounds are methanol (MeOH) and acetonitrile (ACN). Three kinds of solvent system were investigated 100% (*v*/*v*) methanol, 50% (*v*/*v*) methanol/acetonitrile and 100% (*v*/*v*) acetonitrile. From Figure S4B. It can be seen that 100% (*v*/*v*) methanol showed the highest peak area in case of most of the ABs except STZ. Therefore, 100% methanol was selected as an optimum desorption solvent in the following experiments.

### 2.4. Comparison with Commercial Fibers

**Figure 6:**
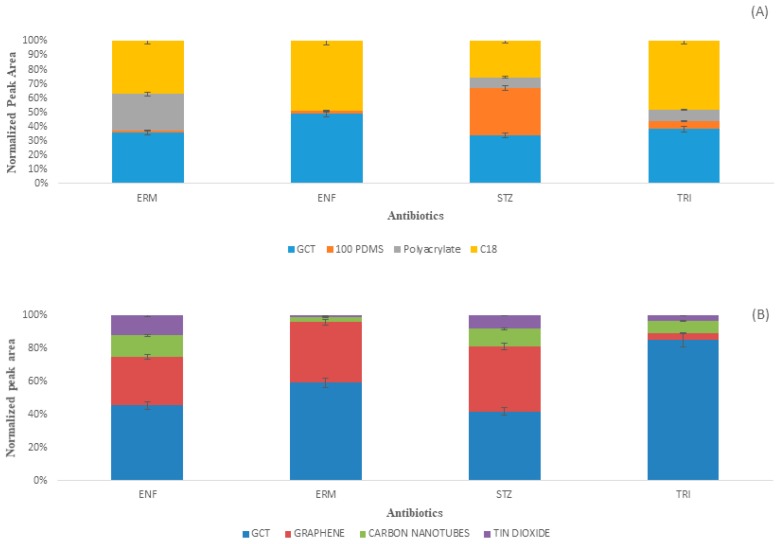
Comparison of extraction efficiency among (**A**) GCT components and with (**B**) different commercial fibers for the extraction of antibiotics.

The GCT fiber was first compared with the individual components of the GCT composite material. The individual component fiber was prepared with PAN in the same way as the preparation of GCT composite fiber. From Figure 6A, it was confirmed that the GCT fiber has more extraction efficiency than the individual components. After confirming its superiority over individual components, it was compared with different commercial fibers (Figure 6B) under its optimized conditions where it was found to be superior to C18, PDMS, or acrylate fibers regarding higher extraction efficiency. In conclusion, the GCT composite has noteworthy adsorptive capacity due to its structure and surface. The comparison of antibiotic detection by different methods has been compared with the present study (Table S2) and mostly found to be superior compared to the existing methods.

### 2.5. Evaluation of Method Performance

To validate the performance of the proposed SPME procedure, a series of experiments regarding the analytical characteristics such as linearity, limit of detection (LOD), limit of quantification (LOQ), intra-batch reproducibility, batch-to-batch reproducibility and accuracy were carried out under the optimal conditions, and the results are listed in Table 1.

The calculated signal to noise ratios of 3 (S/N = 3) and 10 (S/N = 10) signify the limit of detection (LOD) and limit of quantification (LOQ), respectively [54]. The relative standard deviation (RSD) for the intra-fiber reproducibility (*n* = 6) of six sampling-desorption cycles ranged from 1.8% to 6.8%, indicating satisfactory stability for repeated use of the custom-made fiber. Inter-fiber reproducibility was checked by the corresponding RSDs which ranged from 4.5% to 8.8% (*n* = 6), The as-prepared fiber can be well-regenerated by dipping the fiber in methanol for 30 min.

### 2.6. Application in Real Samples

The practical application of the proposed methods was checked by detecting trace amounts of multiple classes of ABs in three water bodies, including river, pond and tap water. Target compounds were measured according to the developed calibration curve (which were developed in milliQ water assuming the absence of matrix). Various concentrations (100.0 and 200.0 ng/L) of each analyte were spiked into the water samples to investigate the matrix effect concerning spiked recoveries. The triplicate study evaluated the reproducibility of the spiked recoveries. From Table 2 it can be seen that the spiked recoveries varied from 81.5% to 113.3% with RSDs of 3.6 to 10.2%. From the results, it can be concluded that there is no significant matrix effect and the proposed method is sensitive and reliable to simultaneously detect and quantify multiple classes of ABs in a complex water system. 

## 3. Materials and Methods

### 3.1. Chemicals and Materials

Enrofloxacin (ERF), sulfathiazole (STZ), erythromycin (ETM), trimethoprim (TMP), multiwalled carbon nanotubes and tin dichloride dihydrate (SnCl_2_·2H_2_O) were purchased from Aladdin Reagent Company (Shanghai, China). Graphene oxide was obtained from J&K Scientific (Beijing, China); analytical grade hydrochloric acid, sodium hydroxide, and methanol were purchased from Guangzhou Reagent Company (Guangzhou, China). Polyacrylonitrile (PAN) and HPLC grade methanol were purchased from Sigma-Aldrich Co. Ltd. (St. Louis, MO, USA). All the chemicals were used without further purification. Quartz fiber (960 µM O.D.) were obtained from Scitlion Technology Co., Ltd (Beijing, China). Throughout the experiment ultrapure water from Merck Millipore (Burlington, MA, USA) was used. A mixture of antibiotic stock solutions (1000 and 10 mg L^−1^) was prepared and stored refrigerated when not in use. The working solution was made with ultrapure water by serial dilution technique before use.

### 3.2. Synthesis of GCT Hybrid Architectures

The synthesis of three-dimensional graphene oxide/carbon nanotube/SnO_2_ hybrid architecture (Figure S5) was performed according to the literature with slight modifications [45]. In brief GO (20 mg) was added to ultrapure water (20 mL) to form a dispersion. CNTs (10 mg) were slowly added into the GO dispersion. The mixture was then ultrasonicated for 40 min. Afterwards, tin dichloride dihydrate (180 mg) was added gradually to form a stable complex solution and stirred for 40 min. The suspension was then sealed in a 25 mL Teflon-lined autoclave and kept for 12 h at 180 °C. After cooling, followed by centrifugation, the precipitate was collected and dried for 10 h at 50 °C. The dried material was grounded in a pestle-mortar to prepare a small sized fine powder.

### 3.3. SPME Fiber Preparation Methods

Quartz fibers (QFs) were cut into a small piece of 3 cm length and sonicated in water (15 min) and methanol (25 min), respectively. Then the top of the polymer was peeled off by a metal fork, and the fiber was collected. The QF surface was neutralized by dipping in 0.1 M sodium hydroxide followed by 0.1 M hydrochloric acid for half an hour each stage. The QFs were dried for 12 h. at room temperature and prepared for coating.

An excellent and equal average particle size are needed to form a uniform coating onto the surface of QFs. Therefore, small-sized fine particles of GCT were dissolved in methanol and ultrasonicated for an hour, followed by drying in an oven at 50 °C.

A glue-like material was prepared to attach the GCT adsorbent onto the surface of QFs. The gluing material preparation process can briefly describe as follows: PAN (100 mg) was dissolved in anhydrous DMF (1 g) in a 2 mL Eppendorf tube. Sonication of the mixture was done for one hour to prepare a yellow colored homogeneous viscous mixture. To optimize the GCT amount, different amounts (25, 50, 75 and 100 mg) of GCT were added to the same concentration of prepared yellow colored viscous material to form a homogenous mixture. The different mixtures were then sonicated for 1 h to form different dispersed slurries. The pretreated QFs were dipped in the different slurries and pulled out gradually so that a uniform layer with a length of 1 cm of GCT adsorbent was attached to the QF surface. This process was repeated several times until a sufficient amount of GCT was attached to the QF surface [29]. The best-optimized adsorbent was chosen according to the uniformity with the highest attachment to the QFs surface (Table S3). The best-optimized amount of GCT attached to the surface of QFs was found to be 50 mg. The self-made fiber now can be used for the quantification of pollutants in the LC-MS/MS instrument.

### 3.4. Instrumentation

TGA analysis was conducted on a TGA 2 apparatus (Mettler Toledo, Columbus, OH, USA) over a temperature range from room temperature to 800 °C at a heating rate of 15 °C min^−1^ and under the air flow of 200 mL min^−1^ to check the thermal stability of the prepared adsorbent.

The X-ray diffraction (XRD) patterns eere collected on a Bruker AXS D8 advance diffraction instrument (Bruker, Karlsruhe, Germany) operating at an accelerating potential of 40 kV, tubes current of 20 mA and using Cu Kα radiation (λ = 1.5406 Å) at room temperature. At first, the GCT samples were ground to form a fine powder and scanned for 2 s per degree from 5−80°, to elucidate the crystalline structure of the samples.

After 10 min of incubation, the effective diameter (nm) and zeta potential (mV) were determined by utilizing a Brookhaven instrument (Holtsville, NY, USA). The presented data based on three independent measurements and given as mean ± standard deviation (SD).

Scanning electron microscopy (SEM) images were recorded to see the surface morphological images of GCT on a Quanta 400 Thermal FE Environment Scanning Electron Microscope instrument (FEI/OXFORD/ HKL, Eindhoven, The Netherlands) using 20 kV.

The synthesized samples functional group and bonds were estimated by using a model IFS 66v/s IR spectrometer (Bruker). X-ray Photoelectron Spectroscopy (XPS) was performed to study the quantitative and chemical nature of the materials using an ESCALAB 250 system (Thermo Fisher Scientific, Waltham, MA, USA) with Cu Kα radiation.

High-performance liquid chromatography-tandem mass spectrometry (MS/MS) was used to measure the concentration of ABs using an Agilent 1260 HPLC system (Agilent Technologies, Santa Clara, CA, USA) attached with an AB Sciex Triple Quad 4500 triple-quadrupole tandem mass spectrometer (Applied Biosystems/MDS Sciex, Framingham, MA, USA) with a dual ion mode (positive and negative) ESI source.

An Agilent Technologies Zorbax SB-C18 column was used to separate the antibiotic compounds in the HPLC system. The specifications of the column were height 150 mm, width 2.1 mm and particle size 3.6 µm. The apparent surface area (SBET) was calculated from the N_2_ isotherms using the Brunauer−Emmett−Teller (BET) method.

### 3.5. Instrumental Analysis

High-performance liquid chromatography-tandem mass spectrometry (MS/MS) instrument was performed on an Agilent 1260 HPLC system (Agilent Technologies) attached to an AB Sciex Triple Quad 4500 triple-quadrupole tandem mass spectrometer with a positive and negative ion mode ESI source. The separation of analytes was done by using a Zorbax SB-C18 column (2.1 mm × 150 mm, 3.6 μm. Heptafluorobutyric acid (0.01%)-containing aqueous solution (A) and methanol (B) were used as mobile phases at flow rates of 300 μL·min^−1^. The initial gradient of 100% A was ramped to 60% A in 4 min and kept for 3.10 min, then ramped back to 55% A in 7.10 min and kept for 2 min, then again ramped back to 25%, 0% and 100% with respect to time kept at 2.9 min, 0.1 min and 5 min respectively to balance the column pressure. All the transitions of ions were monitored as positive ion mode: STZ *m*/*z* 255.9/107.8, TRI *m*/*z* 291/123.1; ENF *m*/*z* 360.1/244.8; and ERM *m*/*z* 734.6/576.3. The optimum conditions for MS/MS analysis of ABs have been depicted in Table 3.

## 4. Conclusions

In this work, a GCT fiber was proposed as SPME fiber for the detection of multiple classes of ABs in aqueous systems. The structural topographical characteristics of GCT powder and its fiber were examined using XRD, TGA, FTIR, XPS, SEM, and TEM. The proposed method showed high sensitivity for most of the analytes and good reproducibility in the extraction of multiple classes of ABs from water. In the detection of ABs in a real environmental sample, this method has shown high recovery with good reproducibility. Moreover, as compared to commercial fiber it showed better sensitivity, lower cost, a wide linear range, and an easy preparation method, therefore, the proposed GCT fiber-based SPME-LC-MS/MS method is shown to be promising for the analysis of antibiotic mixtures in real environmental samples.

## Figures and Tables

**Table 1:** Linear ranges (ng L^−1^), linear regression coefficient (R^2^), LOD (S/N = 3, ng L^−1^), LOQ (S/N = 10, ng L^−1^) and intra-fiber and inter-fiber reproducibility (%) of the custom-made fiber (*n* = 6) in water sample.

Antibiotics	Linear Range (ng L^−1^)	R^2^	LOD (ng L^−1^) S/N = 3	LOQ (ng L^−1^) S/N = 10	Intra-Fiber RSD%	Inter-Fiber (*n* = 6) RSD%
ENF	20–50000	0.999	6.6	12.2	5.1	6.1
STZ	20–50000	0.997	7.69	15.6	6.8	8.8
ETM	10–50000	0.996	1.36	4.54	2.8	4.6
TMP	10–50000	0.998	0.9	3.02	1.8	4.5

**Table 2:** Concentration (µg L^−1^) and recoveries (*n* = 3) of antibiotics at different spiked levels in real environmental samples.

	**River Water**			
**Antibiotics**	**Detected Conc. (ng/L)**	**Spiked Conc. (ng L^−1^)**	**Recovery**	**RSD%**
ENF	46	100 200	92.14 89.38	9.8 8.2
STZ	19.3	100 200	89.3 82.1	7.3 4.6
ERM	47.2	100 200	113.3 104.5	5.8 4.3
TMP	12.93	100 200	89.4 89.6	4.6 4.8
	**Pond Water**			
	**Detected Conc. (ng/L)**	**Spiked Conc. (ng L^−1^)**	**Recovery**	**RSD%**
ENF	9.2	100 200	85.57 87.3	7.9 6.9
STZ	13.42	100 200	82.7 81.5	4.9 6.3
ERM	33.2	100 200	94.45 91.68	7.9 7.2
TMP	7.29	100 200	90.7 90.4	4.1 5.2
	**Tap Water**			
	**Detected Conc. (ng/L)**	**Spiked Conc. (ng L^−1^)**	**Recovery**	**RSD%**
ENF	Not detected	100 200	84.86 82.95	9.7 10.2
STZ	Not detected	100 200	84.40 80.34	8.6 8.3
ERM	29.5	100 200	95.16 89.39	4.2 4.6
TMP	6.26	100 200	91.46 90.24	3.6 4.3

**Table 3:** Optimum conditions for MS/MS (ESI positive mode) analysis of antibiotics (DP: declustering potential; EP: entrance potential; CE: collision energy; CXP: collision cell exit potential. V: Volts. Da: Dalton).

Antibiotics	Molecular Weight	Q1 Mass (Da)	Q3 Mass (Da)	DP (V)	EP (V)	CE (V)	CXP (V)
Enrofloxacin	359.39	360.1	244.8	116	11	33	17
Sulfathiazole	255.32	255.9	107.8	64	12	36	14
Erythromycin	733.93	734.6	576.3	164	12	29	30
Trimethoprim	290.32	291	230	70	7	34	9

## Supplementary Materials

The following are available online.
