# Supplementary material for: Carbon and Tin-Based Polyacrylonitrile Hybrid Architecture Solid Phase Microextraction Fiber for the Detection and Quantification of Antibiotic Compounds in Aqueous Environmental Systems
Source: Molecules. 2019 Apr 28;24(9):1670. doi: 10.3390/molecules24091670 (PMC6539674; doi:10.3390/molecules24091670)
Supplement: Supplementary file 1 [file molecules-24-01670-s001.pdf]

Supporting Information

Carbon and Tin based polyacrylonitrile hybrid architect solid phase microextraction fiber for the detection and quantification of antibiotic compounds from aqueous environmental systems.

Sandip Mondal, Jialing Jiang, Yin Li, Gangfeng Ouyang*

MOE Key Laboratory of Bioinorganic and Synthetic Chemistry/KLGHEI of Environment and Energy Chemistry, School of Chemistry, Sun Yat-sen University, Guangzhou 510275, China.

* Corresponding author: +86-20-84110845; cesoygf@mail.sysu.edu.cn (G. Ouyang).

Page 11

Figure 5

Table 4

Figure S1: Image of (a) effective diameter and (d) zeta potential of GCS.

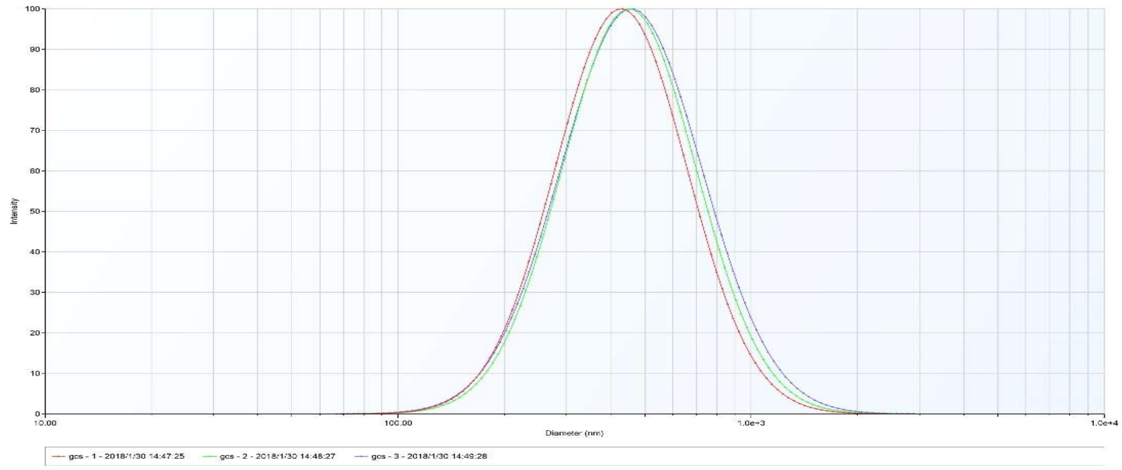

(A)

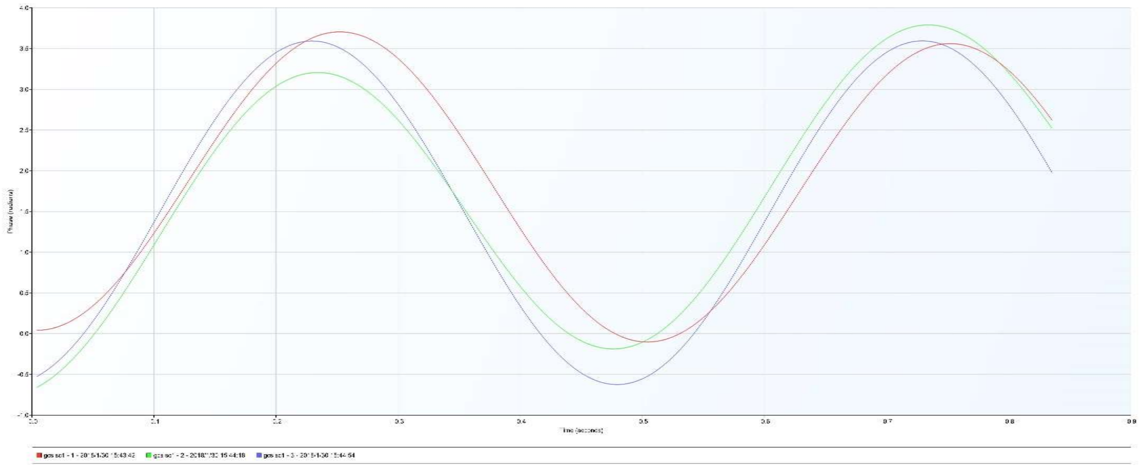

(B)

Figure S2: XPS spectra of GCT.

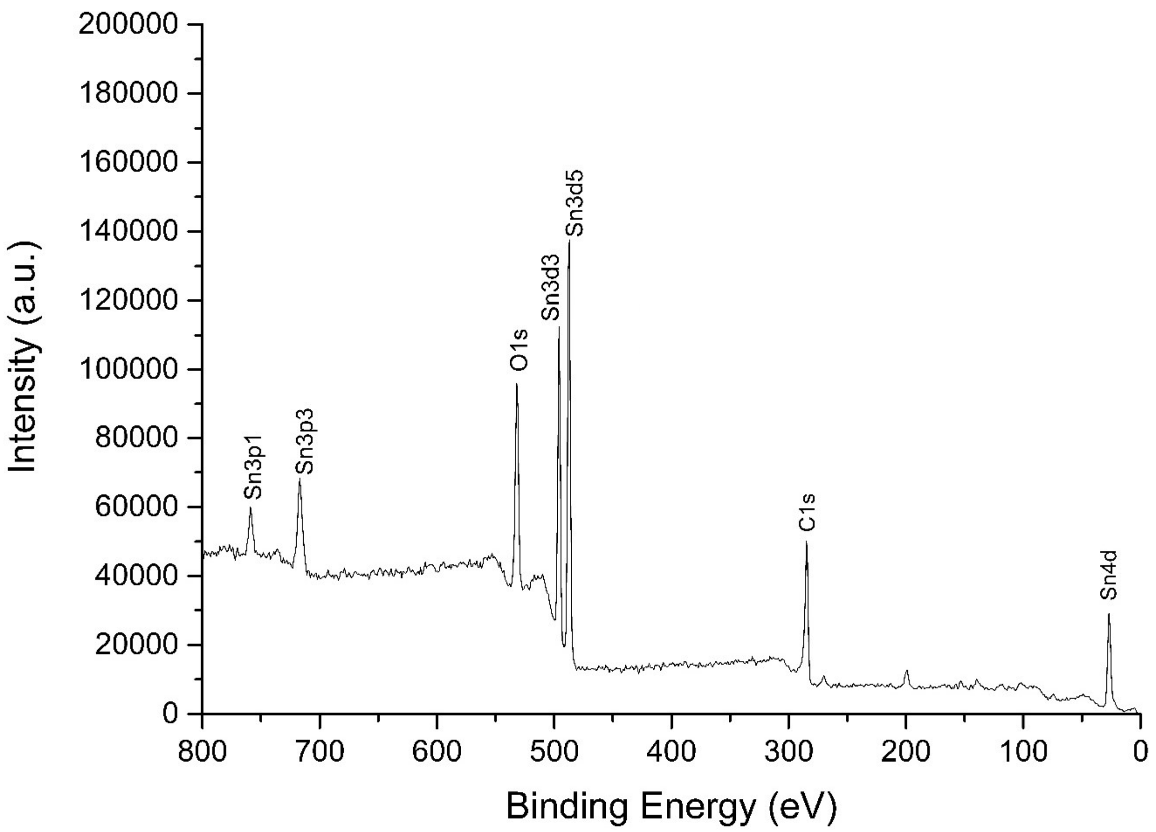

Figure S3. selected area electron diffraction (SAED) pattern of GCT

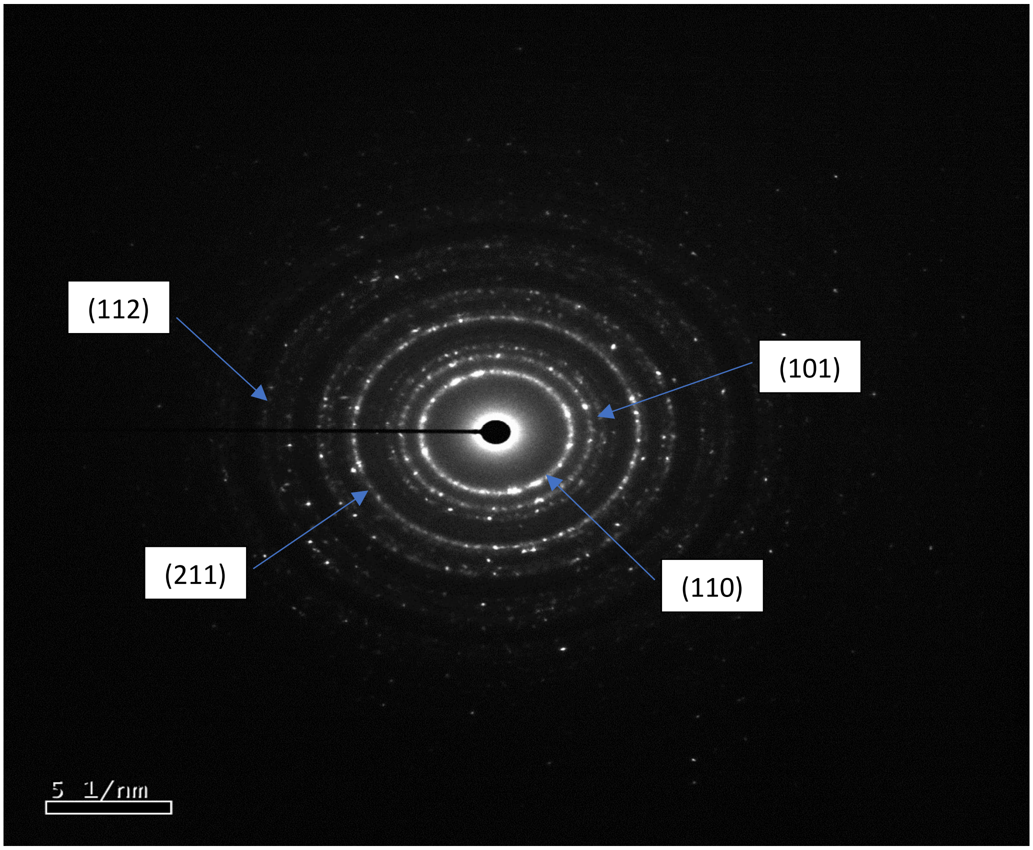

Figure S4. (A) Carry over experiment and (B)Optimization of solvent system.

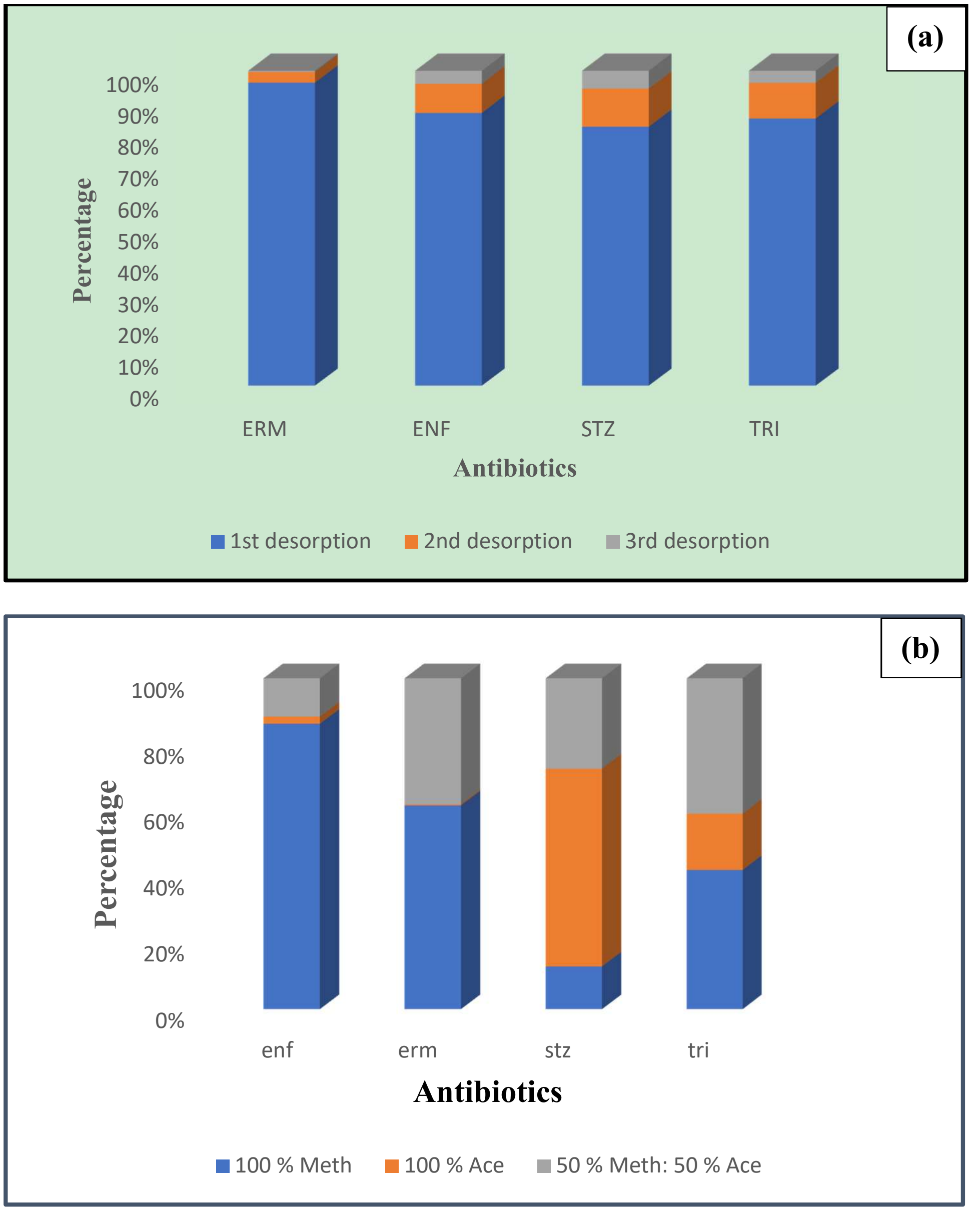

Figure S5: Fabrication of GCT Hybrid Architecture

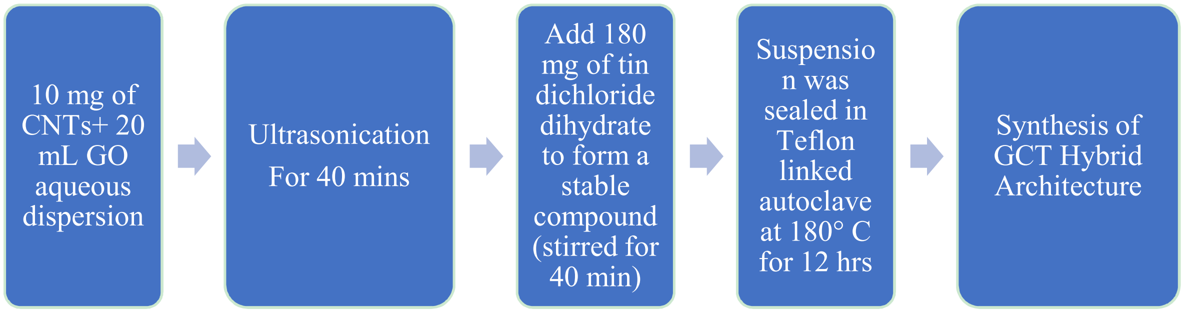

Table S1: Cost analysis to prepare SPME (N=360) fiber.

(A) Solid compound	Amount (gm)	Unit Price (RMB)	amount used (gm)	Price (RMB)		
Carbon nanotubes	1	3078	0.12	369.36		
Graphene oxide	1	1799	0.24	431.76		
Tin dicholoride dihydrate	500	147	2.16	0.63		
Polyacrylonitrile	100	2574	1.2	30.888		
NaOH	500	15	0.2	0.006		
Total cost				832.649		
**(B) Liquid compound**	**Amount (mL)**	**Price (RMB)**	**Used (ml)**	**Price (RMB)**		
DMF	500	26	12	0.62		
Hydrochloric acid	500	19	0.41	0.02		
Total cost				0.64		
**(C) Equipment**	**Ampere (A)**	**Volts (V)**	**Watts (W)**	**Time (hour)**	**Unit cost (RMB)**	**Cost (RMB)**
Centrifuge	40	220	8800	1	0.6	0.6
Sonication	48	230	11040	1.66	0.6	0.36
Hot air oven			320	12	0.6	7.2
Total cost						8.16
**(D) Fiber material**	**Length**	**Unit Price**	**No. of fiber**	**Length of fiber (cm)**	**Length of fiber used (m)**	**Price (RMB)**
Quartz fiber	1 m RMB	120	360	3	10.8	1296
(E) Net Cost	2137.45					
(F) Other overhead cost=10% of the net cost	213.745					
(G) Total cost	2351.2					
**Number of fibers (N)**	**Net cost (RMB)**	**1 Fiber cost (RMB)**				
360	2351.2	6.53				

The break-up cost for each step and the total cost for the preparation of 360 fiber in Chinese Renminbi (RMB) have been calculated stepwise to give an idea about the approximate cost involved in the present investigation. So, the approximate cost of fiber is (Total cost/360 fiber) **6.53 RMB or 0.97 USD** which is much cheaper than the commercial fiber (1000 RMB or 149 USD).

Table S2: Comparison of antibiotic detection by different methods

Antibiotic	Medium	Methods	LOD	References
**Enrofloxacin**	Milk	Glucose meter	5 ng/mL	Kwon et al.(2018) [1]
	Water	DSPE-HPLC	0.36 µg/L	Lu et al (2019)[2]
	Water	MEKC	1 µg/L	Brompoj (2018)[3]
	Water	HPLC-DAD	0.1 mg/L	Danijela (2010)[4]
	Water	SPME-LC-MS/MS	6.6 ng/L	**This study**
**Sulfathiazole**	Water	Immunosensor	0.11 µg/L	Dolors (2010)[5]
	Water	HPLC–MS/MS	0.29 ng/L	Guiju (2019)[6]
	Sea water	MS-MS	1.40 ng/mL	Sara (2015)[7]
	Sea Water	UV	2.88 µg/mL	Sara (2015)[7]
	Water	HPLC-DAD	0.20 µg/L	Kochaporn [8]
	Water	HPLC-DAD	4.48 µg/L	Antonio [9]
	Water	SPME-LC-MS/MS	7.69 ng/L	**This study**
**Erythromycin**	Water	HPLC–MS/MS	0.04 ng/L	Anwar (2018)[10]
	Water	HPLC–MS/MS	0.10 ng/L	Nadia (2017)[11]
	Water	HPLC–MS/MS	0.10 ng/L	Hu (2014)
	Water	HPLC–MS/MS	3 ng/L	Hu (2014) [12]
	Water	HPLC–MS/MS	4 ng/L	Thomas (2004)[13]
	Water	HPLC–MS/MS	10 ng/L	Martin (2003) [14]
	Water	SPME-LC-MS/MS	1.36 ng/L	**This study**
**Trimethoprim**	Water	SPE-LC–MS/MS	3.1 ng/L	J.Rossmann(2014)[15]
	Water	HPLC-DAD	0.5 mg/L	Danijela (2010)[4]
	Water	HPLC–MS/MS	10 ng/L	Martin (2003)[14]
	Water	SPME-LC-MS/MS	0.9 ng/L	**This study**

Table S3: Optimization of adsorbent onto the QFs surface

Attachment of highest amount of adsorbent	Amount of PAN+DMF	Uniformity of slurry
20 mg	1.1 g	Visually uniform
50 mg	1.1 g	Visually uniform
75 mg	1.1 g	Visually not uniform. Too much compound makes the slurry saturated and hard, no more viscous solution
100 mg	1.1 g	Visually not uniform. Too much compound makes the slurry saturated and hard, no more viscous solution

Abbreviations:

DSPE: Dispersive solid-phase extraction
MEKC: micellar electrokinetic chromatography
HPLC-DAD: high-performance liquid chromatography with diode array

References:

1. Kwon, D.; Lee, H.; Yoo, H.; Hwang, J.; Lee, D.; Jeon, S., Facile method for enrofloxacin detection in milk using a personal glucose meter. *Sens. Actuators B Chem.* **2018,** 254, 935-939.
2. Lu, W.; Liu, J.; Li, J.; Wang, X.; Lv, M.; Cui, R.; Chen, L., Dual-template molecularly imprinted polymers for dispersive solid-phase extraction of fluoroquinolones in water samples coupled with high performance liquid chromatography. *Analyst* **2019,** 144, (4), 1292-1302.
3. Prutthiwanasan, B.; Suntornsuk, L., Improved resolution of fluoroquinolones using cetyltrimethyl ammonium bromide–micellar electrokinetic chromatography and its application to residue analysis in surface water. *J. Chromatogr. B* **2018,** 1092, 306-312.
4. Ašperger, D.; Babić, S.; Pavlović, D. M.; Dolar, D.; Košutić, K.; Horvat, A. J. M.; Kaštelan-Macan, M., SPE-HPLC/DAD determination of trimethoprim, oxytetracycline and enrofloxacin in water samples. *Int. J. Environ. Anal. Chem.* **2009,** 89, (8-12), 809-819.
5. Jornet, D.; González-Martínez, M. A.; Puchades, R.; Maquieira, A., Antibiotic immunosensing: Determination of sulfathiazole in water and honey. *Talanta* **2010,** 81, (4), 1585-1592.
6. Xu, G.; Zhang, B.; Wang, X.; Li, N.; Zhao, Y.; Liu, L.; Lin, J.-M.; Zhao, R.-S., Porous covalent organonitridic frameworks for solid-phase extraction of sulfonamide antibiotics. *Microchim. Acta* **2018,** 186, (1), 26.
7. Leston, S.; Nebot, C.; Nunes, M.; Cepeda, A.; Pardal, M. Â.; Ramos, F., Sulfathiazole: Analytical methods for quantification in seawater and macroalgae. *Environ. Toxicol. Pharmacol.* **2015,** 39, (1), 77-84.
8. Chullasat, K.; Nurerk, P.; Kanatharana, P.; Kueseng, P.; Sukchuay, T.; Bunkoed, O., Hybrid monolith sorbent of polypyrrole-coated graphene oxide incorporated into a polyvinyl alcohol cryogel for extraction and enrichment of sulfonamides from water samples. *Anal. Chim. Acta.* **2017,** 961, 59-66.
9. Herrera-Herrera, A. V.; Hernández-Borges, J.; Afonso, M. M.; Palenzuela, J. A.; Rodríguez-Delgado, M. Á., Comparison between magnetic and non magnetic multi-walled carbon nanotubes-dispersive solid-phase extraction combined with ultra-high performance liquid chromatography for the determination of sulfonamide antibiotics in water samples. *Talanta* **2013,** 116, 695-703.
10. Hossain, A.; Nakamichi, S.; Habibullah-Al-Mamun, M.; Tani, K.; Masunaga, S.; Matsuda, H., Occurrence and ecological risk of pharmaceuticals in river surface water of Bangladesh. *Environ. Res.* **2018,** 165, 258-266.
11. Torres, N. H.; de Salles Pupo, M. M.; Ferreira, L. F. R.; Maranho, L. A.; Américo-Pinheiro, J. H. P.; Vilca, F. Z.; de Hollanda, L. M.; Tornisielo, V. L., Spatial and

seasonal analysis of antimicrobials and toxicity tests with Daphnia magna, on the sub-basin of Piracicaba river, SP, Brazil. *J. Environ. Chem. Eng.* **2017,** 5, (6), 6070-6076.

12. Hu, F.-Y.; He, L.-M.; Yang, J.-W.; Bian, K.; Wang, Z.-N.; Yang, H.-C.; Liu, Y.-H., Determination of 26 veterinary antibiotics residues in water matrices by lyophilization in combination with LC–MS/MS. *J. Chromatogr. B* **2014,** 949-950, 79-86.
13. Thomas, K. V.; Hilton, M. J., The occurrence of selected human pharmaceutical compounds in UK estuaries. *Mar. Pollut. Bull.* **2004,** 49, (5), 436-444.
14. Hilton, M. J.; Thomas, K. V., Determination of selected human pharmaceutical compounds in effluent and surface water samples by high-performance liquid chromatography–electrospray tandem mass spectrometry. *J. Chromatogr. A* **2003,** 1015, (1), 129-141.
15. Rossmann, J.; Schubert, S.; Gurke, R.; Oertel, R.; Kirch, W., Simultaneous determination of most prescribed antibiotics in multiple urban wastewater by SPE-LC–MS/MS. *J. Chromatogr. B* **2014,** 969, 162-170.
